# Absolute lymphocyte count as a predictor of *Pneumocystis* pneumonia in patients previously unknown to have HIV

**DOI:** 10.3402/jchimp.v2i1.15696

**Published:** 2012-04-30

**Authors:** Aghogho A. Omene, Robert P. Ferguson

**Affiliations:** Department of Internal Medicine, Union Memorial Hospital, Baltimore, MD, USA

## Abstract

This is a retrospective review of patients admitted to an inner city community hospital with community-acquired pneumonia who were ultimately diagnosed with AIDS and *Pneumocystis*. Absolute lymphocyte count in our hospital is available immediately. In contrast, it can take 48 hours or longer to obtain more specific CD-4 counts and AIDS enzyme-linked immunosorbent assay (ELISA) serology. The association of lymphopenia with ultimate diagnosis of AIDS and *Pneumocystis* supports immediate empiric treatment for pneumocystis carinii pneumonia (PCP) in our highly HIV prevalent hospital.

Although the number of newly diagnosed HIV patients with or without AIDS is declining in North America, the prevalence in Maryland has never been higher, a direct result of increased life expectancy (1) (Fig. 1). As recently as 2009, there were 29,080 HIV-positive patients identified in Maryland, and the assumption is there are many more who are yet to be identified. As Baltimore has approximately half of all of Maryland's HIV-positive cases, our hospital continues to admit many cases of AIDS. Since there is a delay in many hospitals, including ours, in obtaining CD-4 counts, we hypothesized that clinicians could rely on absolute lymphocyte counts to make immediate clinical decisions, i.e., is lymphopenia predictive of pneumocystis carinii pneumonia (PCP) in patients destined to be diagnosed with AIDS?

**Fig. 1:**
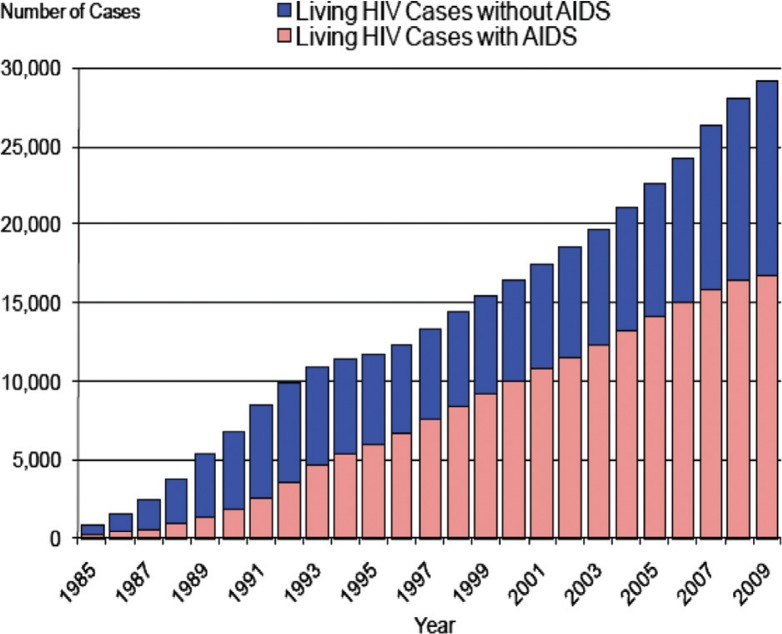
Trends in living cases of HIV in the State of Maryland, 1985–2009

## Methods

We established a database of all HIV-positive admissions to our hospital over a 4-year period, reviewed each individual chart, and set aside the new cases. New AIDS cases were defined as patients non-HIV-positive on admission, who had no prior medical record of being HIV-positive, who were subsequently diagnosed with HIV, and a low CD-4 count. A clinical diagnosis of community-acquired pneumonia was made on admission by history, physical examination, and chest x-ray; *Pneumocystis* was diagnosed by clinical criteria and/or bronchial lavage. Absolute lymphocyte count on admission was considered to be low if measured at 1.0 or less in the initial complete blood count (CBC).

## Results

From 2006 to 2009, there were 2,786 admissions to our hospital who were ultimately identified as HIV positive. Of these, there were 64 new cases of HIV; 40 of which had progressed to AIDS at the time of diagnosis. From these 40 new cases of AIDS, 32 (80%) were ultimately diagnosed with *Pneumocystis* pneumonia. The next highest frequency of AIDS indicating disease was systemic candidiasis (four of the 40 new cases of AIDS had candidiasis). Absolute lymphocyte counts for the 40 newly diagnosed AIDS patients are listed in Table 1. Lymphocyte counts of 1.0 and lower occurred in 27 of the 40 AIDS patients (73%). Of the remaining 13 AIDS patients, seven had an absolute lymphocyte count between 1.0 and 2.0, three had a count >2, and three were unspecified; 23 of the 32 diagnosed with *Pneumocystis* had their lactate dehydrogenase (LDH) levels tested, and their levels were found to be elevated. CD-4 counts arrived at an average of 44 hours post-admission, similar to the HIV enzyme-linked immunosorbent assay (ELISA) test.

**Table 1:** Lymphocyte count of newly diagnosed AIDS patients

Absolute lymphocyte count	Number of AIDS patients
≤1	27 (73%)
1.1–2.0	7
>2	3
Unspecified	3

## Discussion

The attack on CD-4 lymphocytes by HIV leads to disease progression (2). At certain CD4 levels, the risk for AIDS-indicator diseases increases. It has been shown that absolute lymphopenia correlated with PCP in a non-HIV disease (3). Although the lymphocyte count correlates well with declining CD-4 cells and thus progression of disease, we are not aware of recommendations to use this expeditiously on admission, i.e., that with AIDS as a possibility, empiric therapy for PCP should be initiated if the lymphocyte count is low, while the other tests are still pending.

Although HIV prevalence in our hospital remains high, new cases are quite rare. Only 2% of our database was diagnosed for the first time during this time frame, as over a 4-year period, 64 patients were admitted with newly diagnosed HIV. In a patient denying high-risk behavior, early attention to diagnose *Pneumocystis* should nevertheless be pursued because it remains a potentially fatal illness (4), and a delay of antibiotics and steroids of 1 or 2 days can be significant (5). As we have shown here that absolute lymphopenia correlates with lower CD-4 counts, an early identification of community-acquired pneumonia with lymphopenia, particularly with high LDH, should merit consideration for treatment with trimethoprim/sulfamethoxazole and steroids.
